# New Concerns for Neurocognitive Function during Deep Space Exposures to Chronic, Low Dose-Rate, Neutron Radiation

**DOI:** 10.1523/ENEURO.0094-19.2019

**Published:** 2019-08-22

**Authors:** Munjal M. Acharya, Janet E. Baulch, Peter M. Klein, Al Anoud D. Baddour, Lauren A. Apodaca, Eniko A. Kramár, Leila Alikhani, Camillo Garcia, Maria C. Angulo, Raja S. Batra, Christine M. Fallgren, Thomas B. Borak, Craig E. L. Stark, Marcello A. Wood, Richard A. Britten, Ivan Soltesz, Charles L. Limoli

**Affiliations:** 1 Department of Radiation Oncology, University of California, Irvine, California 92697; 2 Department of Neurosurgery, Stanford University, California 94305; 3 Department of Neurobiology and Behavior, University of California, Irvine, California 92697; 4 Department of Environmental and Radiological Health Sciences, Colorado State University, Fort Collins, Colorado 80523; 5 Department of Radiation Oncology, Eastern Virginia Medical School, Norfolk, Virginia 23507

**Keywords:** cognitive dysfunction, electrophysiology, long-term potentiation, low dose-rate, neutrons, space radiation

## Abstract

As NASA prepares for a mission to Mars, concerns regarding the health risks associated with deep space radiation exposure have emerged. Until now, the impacts of such exposures have only been studied in animals after acute exposures, using dose rates ∼1.5×10^5^ higher than those actually encountered in space. Using a new, low dose-rate neutron irradiation facility, we have uncovered that realistic, low dose-rate exposures produce serious neurocognitive complications associated with impaired neurotransmission. Chronic (6 month) low-dose (18 cGy) and dose rate (1 mGy/d) exposures of mice to a mixed field of neutrons and photons result in diminished hippocampal neuronal excitability and disrupted hippocampal and cortical long-term potentiation. Furthermore, mice displayed severe impairments in learning and memory, and the emergence of distress behaviors. Behavioral analyses showed an alarming increase in risk associated with these realistic simulations, revealing for the first time, some unexpected potential problems associated with deep space travel on all levels of neurological function.

## Significance Statement

Simulating the space radiation environment to date has been limited by available technology and restricted by the practicalities of implementing protracted terrestrial-based exposures. Now through the use of a new neutron irradiation facility, capable of simulating the realistic low dose-rates found in deep space, we have uncovered striking neurobehavioral and electrophysiological defects in rodents subjected to continuous (6 month) low dose-rate (1 mGy/d) neutron exposures. This study represents the first to document the significant adverse consequences of space-relevant radiation dose rates on the brain, and points to the heightened risks associated with NASA’s upcoming plans for travel to Mars.

## Introduction

Exposure to the deep space radiation environment poses many potential risks to the health of astronauts, but none may be more concerning than the adverse effects of exposure to energetic charged particles on CNS function. Research from multiple laboratories has now provided convincing evidence that exposure to space relevant fluences of multiple types and combinations of charged particles causes impairments on a range of behavioral tasks administered to rodents (Rabin et al., 2011; Britten et al., 2012; Cherry et al., 2012; Haley et al., 2013; Davis et al., 2015; Parihar et al., 2015c, 2018; Whoolery et al., 2017; Carr et al., 2018). One of the significant caveats to such past studies is the relatively high dose rates with which the radiation was delivered, orders of magnitude higher than that expected to be encountered during deep space travel (i.e., ∼1 mGy/d; Zeitlin et al., 2013; Cucinotta et al., 2014).

Although practical limitations have largely prohibited the acquisition of data collected at space-relevant dose rates, the recent development of a new, low dose-rate neutron facility has now provided the capability to more faithfully simulate those low dose rates. With this capacity, studies can now be conducted to more accurately estimate certain CNS risks to astronauts that will be associated with deep space travel, such as to Mars.

Chronic neutron irradiation closely recapitulates the charged particle risks that astronauts will experience during voyages beyond Earth’s magnetosphere. Although neutrons occur with relatively low fluence in free space relative to other charged particles, galactic cosmic radiation (GCR) collisions with the shielding material of a spacecraft produces a substantial emission of secondary neutrons (Norbury and Slaba, 2014; Nelson, 2016). Importantly, neutrons delivered at very low dose rates from ground-based sources (^252^Cf) possess dose-averaged linear energy transfer (LET) similar to high Z and energy (HZE) particles that are prevalent in GCR. Furthermore, because of the inherent contribution from direct and scattered photons, fast neutrons derived from ^252^Cf decay also simulate the secondary ionizations of delta rays emanating from tracks of heavier high-energy charged particles (Chatterjee and Borak, 2001).

Therefore, to further investigate the biological response to irradiation at space-relevant dose rates, we analyzed the effect of GCR-mimetic neutron exposures on CNS functionality. We find that chronic, low dose-rate neutron irradiation suppresses neuronal excitability within the hippocampus, disrupts network level long-term potentiation and produces long-lasting behavioral deficits in mice. Utilization of one risk modeling approach (kernel density estimation coupled with Numbers Need to Harm) suggests that the incidence of severe impairments in learning and memory, and the emergence of distress behaviors may occur in an unacceptably high percentage of astronauts. Understanding the hazards posed by low dose-rate particle radiation to all levels of the nervous system will enable us to develop improved defensive strategies and countermeasures to facilitate safe human space exploration.

## Materials and Methods

### Neutron irradiation and dosimetry

The neutron irradiation facility consists of ∼53 m^2^ of usable space that is surrounded with concrete shielding. An encapsulated source of ^252^Cf neutron with an activity of 1.6 GBq (80 µgm, delivered March 24, 2017), was housed in a Model 149 panoramic irradiator (JL Shepherd). During irradiations the source was elevated to a position 130 cm above the floor. The initial configuration was designed to provide a total dose rate of 1 mGy per day for up to 900 mice and 60 rats simultaneously. A total of 18 vertical racks were located on an arc ∼180 cm from the source. Each rack can accommodate 10 cages with five mice per cage. The irradiation field at the exposure location consists of direct neutrons from the source and scattered neutrons from the walls and floor. There was also a component of gamma rays emitted from the source and scattered photons. Mixed field dosimetry was performed using tissue equivalent proportional counters (TEPC) to measure neutrons and both a miniature Geiger–Muller counter and CaF_2_ thermoluminescent dosimeters to measure photons. Data from the TEPC provides estimates of the spatial dose rate from neutrons to mice as well as the patterns of energy deposition in volumes of tissue similar to the size of a mammalian cell (i.e., lineal energy).

### Animals and irradiations

All animal experimentation procedures described in this study are in accordance with the guidelines provided by NIH and approved by all Institutional Animal Care and Use Committees. Animals were maintained in standard housing conditions (20 ± 1°C; 70 ± 10% humidity; 12 h light/dark cycle) and provided *ad libitum* access to standard rodent chow and water.

A single cohort of 8-month-old, wild-type, male mice (C57BL/6J, The Jackson Laboratory) were divided into two experimental groups, neutron-irradiated and concurrent controls (*N* = 40 mice per group). The mice received a prolonged 6 month (180 d) radiation exposure at a recently established neutron irradiation facility. The facility was equipped with a ^252^Cf neutron source, where a total dose rate of ∼1 mGy/d was delivered with a neutron contribution of 80% of the total dose per day and photons contribute 20% at an approximate distance of 1.8 m from the source. The daily exposure time required to deliver 1 mGy/d ranged from 15.9 to 18.2 h to accommodate for radioactive decay of the source (*T*_½_ = 2.65 y). The source was retracted into the shield for the remaining time in a day to allow personnel access for animal husbandry. Concurrent control mice were similarly housed in an adjacent facility. Following the 6 month exposure period, mice were shipped to respective institutions for follow-up studies.

### Whole cell electrophysiology

At 6 months following completion of neutron irradiation, mice were deeply anesthetized and rapidly decapitated. Brains were immediately immersed in ice-cold cutting solution containing the following (in mm): 85 NaCl, 75 sucrose, 25 glucose, 24 NaHCO_3_, 4 MgCl_2_, 2.5 KCl, 1.25 NaH_2_PO_4_, 0.5 CaCl_2_. Coronal slices (300 µm) containing the hippocampus were prepared using a Vibratome (VTS1200, Leica Biosystems). Brain slices were then incubated in 35°C cutting solution for 1 h. Before recording, brain slices were transferred to aCSF consisting of (in mm): 126 NaCl, 26 NaHCO_3_, 10 glucose, 2.5 KCl, 2 MgCl_2_, 2 CaCl_2_, 1.25 NaH_2_PO_4_. All solutions were equilibrated with 95% O_2_/5% CO_2_.

Intracellular recordings were performed in a submerged chamber perfused with oxygenated aCSF at 2.5 ml/min and maintained at 33°C by a chamber heater (BadController V, Luigs and Neumann). Hippocampal neurons were visualized using DIC illumination on an Olympus BX61WI microscope (Olympus Microscopy) with a CCD camera (C7500, Hamamatsu). Recording pipettes were pulled from thin-walled borosilicate capillary glass (King Precision Glass) using a P97 puller (Sutter Instruments) and were filled with (in mm): 126 K-gluconate, 10 HEPES, 4 KCl, 4 ATP-Mg, 0.3 GTP-Na, 10 phosphocreatine, pH adjusted to 7.3 with KOH, osmolarity 290 mOsm. Pipettes had a 2.5–5 MΩ tip resistance.

Whole-cell recordings were performed on CA1 superficial layer pyramidal neurons in the dorsal hippocampus. Recordings were excluded for neurons with a resting membrane potential (RMP) >−50 mV or where the series resistance increased by >20% of baseline. Pipette capacitance was neutralized for all recordings. Firing properties were assessed during injected current steps (−200 to 750 pA, 1 s). Action potential threshold was measured as the voltage where dV/dt exceeded three times its SD and afterhyperpolarization was measured relative to threshold. Width was the time an action potential, resampled at 100 kHz, exceeded the half-height between threshold and peak voltages. Action potential properties were only measured in the first spike evoked by a depolarizing current for each neuron. Input resistance was calculated from the change in steady-state membrane potential resulting from hyperpolarizing current injections, whereas sag was measured as the difference between the steady-state and peak negative potential during a −100 pA hyperpolarizing current injection. Spontaneous EPSC (sEPSC) activity was measured as inward currents while neurons were voltage-clamped at −65 mV and events were detected in Clampfit (Molecular Devices) by a blinded investigator. sEPSC rise time was the time required to increase from 10 to 90% of peak amplitude and charge transfer was the integrated current of each sEPSC. Events with a rise time >7.5 ms, a peak amplitude of <3 pA or a charge transfer of <25 pC were excluded, along with recordings where the series resistance increased by >20% of baseline.

Data were acquired in pClamp software (Molecular Devices) using a MultiClamp 700B amplifier (Molecular Devices), low-pass filtered at 2 kHz, and digitized at 10 kHz (Digidata 1440A, Molecular Devices). Data analysis was performed using Clampfit and custom written Python scripts. *N* = 8 control mice yielded recordings from *N* = 29/24 cells (for intrinsic and synaptic properties, respectively). Likewise, *N* = 7 neutron-irradiated mice yielded recordings from *N* = 28/25 cells (for intrinsic and synaptic properties, respectively).

### Extracellular field recordings

Hippocampal slices were prepared as previously described (Vogel-Ciernia et al., 2013). Following isoflurane anesthesia, mice were decapitated and the brain was quickly removed and submerged in ice-cold, oxygenated dissection medium containing the following (in mm): 124 NaCl, 3 KCl, 1.25 KH_2_PO_4_, 5 MgSO_4_, 0 CaCl_2_, 26 NaHCO_3_, and 10 glucose. Coronal hippocampal slices (320 µm) were prepared using a Leica vibrating tissue slicer (model VT1000S) before being transferred to an interface recording chamber containing preheated artificial CSF (aCSF) of the following composition (in mm): 124 NaCl, 3 KCl, 1.25 KH_2_PO_4_, 1.5 MgSO_4_, 2.5 CaCl_2_, 26 NaHCO_3_, and 10 glucose and maintained at 31 ± 1°C. Slices were continuously perfused with this solution at a rate of 1.75–2 ml/min while the surface of the slices were exposed to warm, humidified 95% O_2_/5% CO_2_. Recordings began following at least 2 h of incubation.

Field EPSPs (fEPSPs) were recorded from CA1b stratum radiatum apical dendrites using a single glass pipette filled with 2 m NaCl (2–3 MΩ) in response to orthodromic stimulation (twisted nichrome wire, 65 µm diameter) of Schaffer collateral-commissural projections in CA1 stratum radiatum. Pulses were administered at 0.033 Hz using a current that elicited a 50% maximal spike-free response. After establishing a 10–20 min stable baseline, long-term potentiation (LTP) was induced by delivering a single episode of 5 “theta” bursts, with each burst consisting of four pulses at 100 Hz and the bursts themselves separated by 200 ms [i.e., theta-burst stimulation (TBS)]. The stimulation intensity was not increased during TBS.

Coronal slices (1.70–1.98 mm anterior to bregma) from the ventral medial prefrontal cortex (300 µm) were prepared as described (Vogel-Ciernia et al., 2013). Field recordings were obtained by placing a bipolar stimulation electrode (FHC; 25 µm diameter) within cortical layer IV and a glass recording electrode in layer III (Kirkwood and Bear, 1994). LTP was induced by delivering 3–5 trains of 5 theta bursts at 0.05 Hz. The stimulation intensity was not increased during TBS. Data were collected and digitized by NAC 2.0 Neurodata Acquisition System (Theta Burst) and stored on a disk.

Data in the text are presented as mean ± SD, whereas in the figures as mean ± SEM. The fEPSP slope was measured at 10–90% fall of the slope and data in figures on LTP were normalized to the last 10 min of baseline. Electrophysiological measures were analyzed using a Student’s *t* test, *p* < 0.05.

### Behavioral testing

Concurrent behavioral testing of control and irradiated mice (*N* = 14 per group) occurred across 4 weeks, beginning 3 months after the conclusion of chronic neutron irradiation (6 months). A separate cohort (*N* = 8 per group) was administered social interaction testing. An investigator blinded to the experimental cohorts performed all behavioral testing.

*Social interaction testing*. Social interaction and social avoidance behaviors were evaluated in mice using established protocols (Winslow, 2003; Sato et al., 2013a; Gunaydin et al., 2014). Mice were initially each individually habituated to the well-lit (915 lux) test arena (30 × 30 cm) for 2 d (15 min/d). On the third day of the trial, the test mouse was allowed to explore freely for 10 min, before a novel mouse (3-month-old C57BL/6J male, weighing less than the test mouse) being introduced into the arena. The mice were allowed to explore and interact freely without barrier for 10 min, and active interaction or avoidance was recorded. Social interactions included any time the test mouse spent sniffing while in active contact with the novel animal’s snout, flank, or anogenital area, mutual grooming, or directed pursuit of the novel mouse. Concurrently, avoidance behavior was characterized as time the test mouse spent actively avoiding social interactions initiated by the novel mouse.

*Episodic and spatial memory testing*. Novel object recognition (NOR) and object in place (OiP) spontaneous exploration tasks rely on intact hippocampal, medial prefrontal cortex, and perirhinal cortex function (Barker et al., 2007; Barker and Warburton, 2011). The NOR task evaluates episodic recognition memory through measuring the preference of mice to investigate novel environmental changes, whereas the OiP task evaluates associative recognition memory. Both tasks were conducted as described previously (Parihar et al., 2015c). Briefly, NOR and OiP testing occurred in a dimly lit (48 lux) test arena (30 × 30×30 cm) with a layer of fresh bedding that was filmed from above. All bedding was replaced and the arena was thoroughly cleaned with 70% ethanol between trials. For the NOR task, mice were initially habituated to the empty arena for 3 d (10 min/d). The following testing day, two plastic objects (differing in color, shape and size) were magnetically affixed 16 cm apart in the arena and the mouse was allowed 5 min to explore the objects. The mouse was returned to the home cage for 5 min while one familiar object was substituted for a novel object (both objects were cleansed with 70% ethanol). The mouse was then returned to the arena for 5 min of further exploration. OiP testing began 1 week after the NOR task, with 2 d (10 min/d) of habituation. On the third day, mice explored an arena with four unique objects for 5 min before briefly returning to their home cage (5 min). All objects were cleansed with 70% ethanol and the location of two objects was swapped before the mouse was returned for 5 more minutes of exploration. Video of both tasks was scored for time spent interacting (nose within 2 cm) with familiar versus novel (or relocated) objects. The discrimination index was than calculated for each mouse from these values:

[(novel/total exploration time)–(familiar/total exploration time)]×00.

*Anxiety- and depression-like behavior testing*. After completion of NOR and OiP testing, mice were evaluated for anxiety-like behavior with the light–dark box (LDB) test and depressive-like behavior with the forced swim test (FST), using established methods (Bourin and Hascoët, 2003; Petit-Demouliere et al., 2005; Parihar et al., 2018). The LDB arena consisted of a light compartment (30 × 20×27 cm, 915 lux) connected to a dark compartment (15 × 10×27 cm, 4 lux) via a small opening (7.5 × 7.5 cm). Thus, the LDB test contrasts the natural propensity of mice to explore new environments with their degree of anxiety to be in a well-lit space. Mice were placed in the arena for 10 min, and we recorded time spent in each chamber and number of transitions between compartments. In the FST task, we evaluated hippocampal-dependent, depression-like, behavior by measuring the responses of mice to being placed in a tank of water (15 cm diameter × 20 cm, 24°C) for 5 min. We quantified the amount of time each mouse spent floating (despair-like behavior) versus swimming or climbing.

*Fear extinction testing*. To test whether mice could learn and later extinguish conditioned fear responses, we performed a series of established fear extinction (FE) assays as adapted (Cain et al., 2003). Testing occurred in two similar contexts within a behavioral conditioning chamber (17.5 × 17.5 × 18 cm, Coulbourn Instruments) with a steel slat floors (3.2 mm diameter slats, 8 mm spacing). For Context A the chamber was scented with a spray of 10% acetic acid in water, whereas in Context B the steel floor of the chamber was covered with white plastic and a spray of 10% almond extract in water was applied. Initial fear conditioning was performed in Context A after mice were allowed to habituate to the chamber for 2 min. Three pairings (spaced by 120 s) of an auditory conditioned stimulus (CS; 16 kHz tone, 80 dB, lasting 120 s), co-terminating with a foot-shock unconditioned stimulus (US; 0.6 mA, 1 s) were presented. On the following 3 d of extinction training, mice were initially habituated to Context B for 2 min before being presented with 20 non-US reinforced CS tones (16 kHz, 80 dB, lasting 120 s, at 5 s intervals). On a final day of fear testing mice were presented with only three non-US reinforced CS tones (16 kHz, 80 dB, lasting 120 s) at a 2 min intertrial interval in Context B. Freezing behavior was recorded with a camera mounted above the chamber and scored by an automated, video-based, motion detection program (FreezeFrame, Coulbourn Instruments). FreezeFrame algorithms calculate a motion index for each frame of the video, with higher values representing greater motion. An investigator blinded to the experimental groups set the motion index threshold representing immobility for each animal individually, based on identifying a trough separating low values during immobility and higher values associated with motion. Motion index thresholds were set between 10 and 16 for all animals (0 cGy: 10.51 ± 1.86, 18 cGy: 16.1 ± 0.94). Freezing behavior was defined as continuous bouts of 1 s or more of immobility. The percentage of time each mouse spent freezing was then calculated for each phase of the fear response testing.

### Statistical analyses

The level of significance for behavioral testing was assessed by Mann–Whitney’s two-tailed, nonparametric *t* test using Prism data analysis software v6.0. Analysis of fear extinction data were conducted by repeated-measures ANOVA. Given that the distribution of behavior data are gamma distributed, we also applied a nonparametric approach, kernel density estimation (KDE), to calculate the proportion of mice that exhibited impaired performance within each cohort for a given behavior task. Data were analyzed using the Excel add-in and Minitab KDE macro (AMC Kernel density ver 1.0e) available in AMC Software (Royal Society of Chemistry, www.rsc.org/amc/). This algorithm has been designed to estimate population density functions without making any assumptions. It does not use a fixed bin width, but instead calculates an optimal bin width based on the interquartile range. Full details can be found in the technical report (Royal Society of Chemistry, 2001). This algorithm is robust and ideally suited to analyzing non-uniformly distributed data populations. Analogous to the *Z*-score principle, a performance level that encompassed 95% of the probability density of the control cohort’s performance data were selected as the threshold level, below which an individual animal’s performance would be classified as “severely impaired”. We define the performance metric level within the sham cohort that encompasses the top 95% of performers, and thus establish the 5th percentile. We then determine how many of the irradiated mice have performance metrics that fall within the 5th percentile of the sham population performance. To account for the nested data produced by whole-cell electrophysiology recordings, differences between treatment groups were evaluated by a mixed linear model regression analysis (Aarts et al., 2014) run in Python. Calculation of estimation statistics-based confidence intervals (CIs) was performed with the DABEST package in python (Ho et al., 2019). Gardner–Altman estimation plots include a 5000 resampling, bias-corrected, and accelerated bootstrap analysis to determine the nonparametric confidence interval of differences between groups. We quantified effect sizes with an unbiased Cohen’s *d* test. *p* values ≤ 0.05 were considered to be statistically significant.

## Results

The goal of our study was to identify risks to the nervous system associated with chronic exposures to low dose and dose rate neutron radiation, with a specific emphasis on the hippocampus/medial prefrontal cortex neural circuits. We used electrophysiological recordings to identify cellular-level alterations in hippocampal neuron function and associated disruptions in synaptic plasticity. Through behavioral testing, we probed whether deficits likewise occurred in tasks dependent on either the hippocampus or a broader set of brain regions. Collectively, our experimental and risk modeling approaches identify a wide range of hazards associated with chronic particle radiation exposures occurring at all levels of nervous system function.

### Radiation dosimetry

The goal was to deliver a space-relevant dose rate of 1 mGy/d (Zeitlin et al., 2013; Cucinotta, 2014) while having sufficient time in the day to allow personnel access for animal husbandry and maintenance. Neutron dosimetry was performed with tissue equivalent counters. These data were used to assess the spatial dose rates to mice in the room as well as characterize the microdosimetric lineal energy (*y*) and LET (L) (Table 1). Photon dosimetry was performed independently using a neutron insensitive GM counter and CaF_2_ thermoluminescent dosimeters. All dosimetric measurements were made in the room containing the full complement of racks and cages filled with food, water, and 28 g mouse phantoms. The final arrangement of cages and racks immediately before the start of our trial yielded an initial total dose rate of 0.063 ± 0.0063 mGy/hr, emitting the target dose of 1 mGy/d over 15.9 h. Furthermore, we targeted a dose rate that would account for the decay rate of the ^252^Cf source over the course of our trial. Therefore, the time of irradiation each day increased slightly throughout the experiment. By the final day of our 6 month trial, mice were receiving an absorbed dose of 1 mGy/d during an exposure time of 18.3 h/d. The mixed field dosimetric assessment indicated that photons emitted from the source and those originating in the walls and floor made a 20% contribution to the total dose rate throughout the room. The mice were not constrained and were free to move about within their cages; we estimate a mouse to mouse variation of ± 20% in our evaluation of the total absorbed dose.

**Table 1.** Mean values of lineal energy (y) and LET (L) based on frequency (f) and dose (D) of energy deposition events

**Neutrons**	**keV/µm**
${\bar{y}}_{f}$	45
${\bar{L}}_{f}$	46
${\bar{y}}_{D}$	80
${\bar{L}}_{D}$	68
**Total**	**keV/µm**
${\bar{y}}_{D}$	64

### Neutron irradiation suppresses excitatory hippocampal neuronal activity

Past studies on the impacts of acute cosmic radiation exposure have frequently demonstrated significant alterations within the hippocampus, a structure known to play a key role in learning and memory. These include disruptions in the morphology (Parihar et al., 2015a; Carr et al., 2018), intrinsic excitability (Sokolova et al., 2015) and synaptic signaling properties (Sokolova et al., 2015; Lee et al., 2017) of hippocampal neurons. Therefore, we decided to begin by evaluating whether chronic, low dose and dose rate exposures to neutron radiation likewise produce cellular-level deficits in hippocampal function. To account for the nested data produced by whole-cell electrophysiology recordings, differences between treatment groups were evaluated by a mixed linear model regression (MLM) analysis (Aarts et al., 2014). In addition to using statistical inference analysis derived *p* values, we also include estimation statistics-based confidence intervals (Ho et al., 2019).

**Figure 1.**
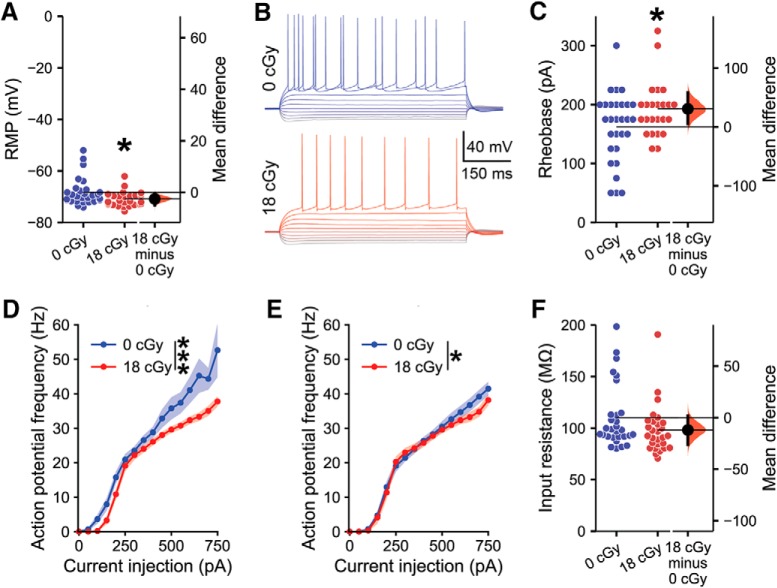
Neutron irradiation alters the electrophysiological properties of CA1 pyramidal neurons. All data are from whole-cell current-clamp recordings of CA1 pyramidal neurons from the superficial layer of the dorsal hippocampus, 6 months following the completion of 18 cGy neutron irradiation. ***A***, RMP was decreased following neutron irradiation. ***B***, Representative examples of responses to a range of brief current injections in neurons from 0 cGy control and 18 cGy mice. ***C***, The rheobase current required to evoke an action potential was greater in neutron-irradiated animals compared with concurrent controls. ***D***, Action potential frequency was attenuated across a range of current injections in 18 cGy neurons, (***E***) including in a subset of neurons with an RMP within −69.6 ± 3 mV. ***F***, There was no significant alteration in input resistance. *N* = 8/7 animals, 29/28 cells (0 cGy and 18 cGy, respectively), except for ***E***, where *N* = 23/16 cells. Gardner–Altman estimation plots show raw data on the left axis and a bootstrapped sampling distribution on the right axis. A black dot depicts the mean difference between groups and the 95% CI is indicated by the ends of the vertical black bars. Data are presented as mean ± SEM for ***D*** and ***E***. *p < 0.05, ***p < 0.001 (MLM regression or two-way ANOVA).

We find that exposure to a protracted 18 cGy dose of neutron radiation produces multiple changes in the intrinsic electrophysiological properties of CA1 superficial layer pyramidal neurons in the dorsal hippocampus that persist at 6 months after the completion of irradiation (Fig. 1). The RMP of neutron-irradiated pyramidal neurons becomes moderately more hyperpolarized than that of control neurons [mean difference score (M_diff_) =−2.56 mV, 95% CI (−5.04, −0.68); *d*=−0.60, 95% CI (−0.97, −0.10); MLM *z*=−2.24, *p* = 0.025; Fig. 1*A*). To further test for changes in cell-intrinsic properties of CA1 pyramidal neurons from neutron-irradiated and control mice, cells were subjected to a range of brief current injections (Fig. 1*B*). Neutron irradiation produces a medium effect-size decrease in the excitability of CA1 pyramidal neurons, where higher rheobase currents are required to evoke action potentials than in neurons from control animals [M_diff_=30.8 pA, 95% CI(5.6, 58.6); *d* = 0.59, 95% CI(0.06, 1.04); MLM *z* = 2.23, *p* = 0.026; Fig. 1*B*,*C*]. Across a range of current injections from 0 to 750 pA, neurons from neutron-irradiated mice exhibit reduced action potential firing frequencies (*F*_(1852)_ = 38.65, *p* < 0.001, two-way ANOVA; Fig. 1*D*). To determine whether action potential firing frequency differences resulted solely from previously noted RMP differences, we examined responses in a subset of neurons that rested within ±3 mV of the overall mean RMP of −69.6 mV. Although more similar, evoked action potential firing remains attenuated in neutron-irradiated versus control mice even when accounting for differences in RMP (*F*_(1574)_ =4.29, *p* = 0.039, *N* = 23 0 cGy cells and 16 18 cGy cells, two-way ANOVA; Fig. 1*E*). The underlying cause of the decrease in RMP we observe remains unclear, but estimation statistics indicate a small decrease in the input resistance of neutron-irradiated neurons [M_diff_ =−12.0 MΩ, 95% CI (−26.4, 1.7); *d* = −0.43, 95% CI (−0.89, 0.14); Fig. 1*F*]. However, variability in measured input resistances resulted in a MLM regression *p* value above traditional significance levels (MLM *z* = −1.63, *p* = 0.103). Likewise, neutron-irradiation does not alter other intrinsic properties of CA1 neurons including action potential threshold, height, width, after polarization or hyperpolarization sag (Table 2). Thus, the combined decrease in RMP and increased rheobase current of cells in the neutron-irradiated brain suggests that chronic exposure to cosmic radiation suppresses the intrinsic excitability of hippocampal pyramidal neurons.

**Table 2.** Neutron radiation-induced alterations in action potential intrinsic properties

**Parameter**	**Mean (95% CI)**		**Mean difference (95% CI)**	**Cohen’s *d*(95% CI)**	**MLM *z* value**	**MLM*p* value**
**0 cGy**	**18 cGy**					
**AP threshold, mV**	−41.9 (−43.2, −40.5)	−41.6 (−43.5, −39.7)	0.29 (−1.74, 2.13)	0.08 (−0.45, 0.67)	0.300	0.765
**AP height**, **mV**	87.5 (82.7, 92.3)	87.9 (81.0, 94.7)	0.37 (−6.05, 7.85)	0.03 (−0.48, 0.57)	0.106	0.915
**AP width**, **ms**	1.24 (1.14, 1.35)	1.19 (1.04, 1.35)	−0.048 (−0.228, 0.081)	−0.17 (−0.64, 0.36)	−0.618	0.536
**AP afterhyperpolarization**, **mV**	−8.45 (−9.84, −7.06)	−7.43 (−9.38, −5.48)	1.02 (−0.75, 3.08)	0.27 (−0.26, 0.72)	1.022	0.307
**Hyperpolarization sag**, **mV**	1.90 (1.62, 2.19)	1.92 (1.51, 2.34)	0.020 (−0.321, 0.366)	0.03 (−0.52, 0.56)	0.134	0.894

CIs for mean values are determined based on an MLM test; CIs for mean difference and Cohen’s *d* are based on a 5000 resampling, bias-corrected, and accelerated bootstrap analysis.

**Figure 2.**
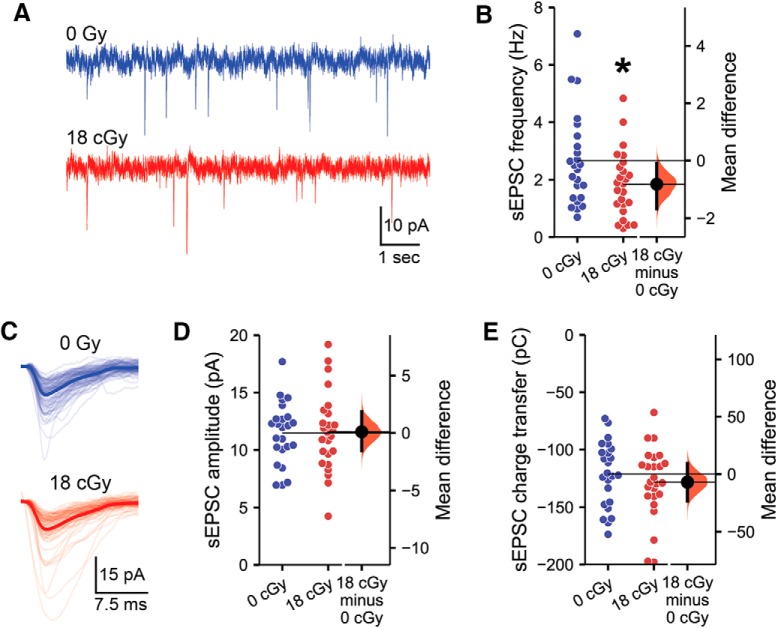
Excitatory synaptic signaling to CA1 pyramidal neurons is decreased following neutron irradiation. All data are from whole-cell recordings of CA1 pyramidal neurons from the superficial layer of the dorsal hippocampus voltage-clamp at −65 mV, 6 months following the completion of 18 cGy neutron irradiation. ***A***, Representative examples of recordings containing spontaneous EPSCs from neurons from neutron-irradiated animals. ***B***, sEPSCs were less frequent in neurons from neutron-irradiated animals. ***C***, Examples of EPSCs in representative neurons from neutron-irradiated animals. Light lines show individual sEPSCs, whereas the darker line displays the average sEPSC during a 200 s recording from that neuron. Neither the sEPSC amplitude (***D***) nor sEPSC charge transfer (***E***) was altered by neutron irradiation. *N* = 8/7 animals, 24/25 cells (0 cGy and 18 cGy, respectively). Gardner–Altman estimation plots show raw data on the left axis and a bootstrapped sampling distribution on the right axis. A black dot depicts the mean difference between groups and the 95% CI is indicated by the ends of the vertical black bars. *p < 0.05 (MLM regression).

Furthermore, the spontaneous, excitatory, postsynaptic activity received by CA1 pyramidal neurons is also altered by exposure to neutrons (Fig. 2). CA1 pyramidal neuron sEPSCs were recorded as inward currents in neurons voltage-clamped at −65 mV. We observe a medium-sized decrease in sEPSC frequency following neutron irradiation relative to in control animals [M_diff_ = −0.81 Hz, 95% CI (−1.67, −0.10); *d*=−0.59, 95% CI (−1.11, −0.002); MLM *z* =−2.03, *p* = 0.043; Fig. 2*A*,*B*]. Thus, there appears to be reduced signaling from presynaptic neurons onto CA1 pyramidal neurons. To examine differences in the sEPSC properties of individual neurons, all sEPSCs detected within a 200 s recording period from each cell were averaged together to generate a standard profile (Fig. 2*C*). Whereas the amplitude of sEPSCs remains equivalent between neutron-irradiated and control animals [*M*_diff_ = 0.09 pA, 95% CI (−1.55, 1.84); *d* = 0.03, 95% CI (−0.56, 0.59); MLM *z* =−0.01, *p* = 0.992; Fig. 2*D*], neutron irradiation produces a small increase in sEPSC charge transfer relative to control cells [M_diff_ =−6.9 pC, 95% CI(−23.3, 9.3); *d* = −0.23, 95% CI(−0.78, 0.35); Fig. 2*E*]. Although high variability in measured sEPSC charge transfer resulted in a MLM regression *p* value above traditional significance levels (MLM *z*= −2.03, *p* = 0.043), a small effect-size decrease perhaps demonstrates a compensatory response to the reduced frequency of excitatory inputs.

The dual reductions in intrinsic excitability and frequency of sEPSCs received by CA1 pyramidal neurons indicate that chronic neutron irradiation suppresses excitatory signaling within the hippocampus. Although not addressed in the current study, acute delivery of similar radiation doses can disrupt hippocampal signaling through altering cell signaling pathways (Lee et al., 2017), voltage-gated membrane currents (Sokolova et al., 2015), neurotransmitter receptor expression (Carr et al., 2018), or neuronal morphology (Parihar et al., 2015a; Carr et al., 2018). Having identified neuron-level changes in hippocampal signaling, we next looked to assess whether chronic neurons irradiation perturbed synaptic plasticity.

### Hippocampal and cortical synaptic plasticity is perturbed by neutron irradiation

Synaptic plasticity enables activity-dependent regulation of the intricately interconnected networks of excitatory neurons and diverse GABAergic interneurons within the hippocampus. Synaptic inputs from CA3 to CA1 pyramidal neurons through the Schaffer collateral pathway have long been known to attenuate with increased activity, known as LTP, and are thought to represent a cellular basis of memory (Schwartzkroin and Wester, 1975; Lynch, 2004). As we observed deficits in cellular-level synaptic signaling among hippocampal neurons following neutron irradiation, we now sought to examine whether LTP was perturbed in either the hippocampus, or in the prefrontal cortex.

**Figure 3.**
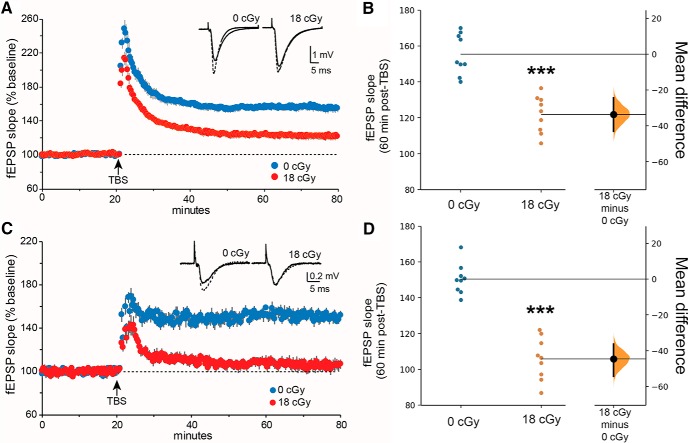
Neutron irradiation alters long-term synaptic plasticity in the hippocampal area CA1 and ventral medial prefrontal cortex. ***A***, ***B***, Extracellular field recordings following stimulation of the Schaffer-commissural projections to the proximal apical dendrites of field CA1b of the dorsal hippocampus, 6 months following completion of the 18 cGy neutron irradiation. ***A***, Following a stable 20 min baseline recording, a single train of TBS was applied and baseline recordings were resumed for an additional 60 min. The time course shows that TBS-induced LTP was markedly reduced in slices from irradiated mice compared with slices from 0 cGy control mice. Inset, Representative traces collected during baseline (solid line) and 60 min post-TBS (dotted line). ***B***, Field EPSP slope was significantly reduced 60 min post-TBS in slices from the neutron-irradiated mice compared with controls. ***C***, ***D***, Field responses recorded in cortical layer III following stimulation of glutamatergic inputs in cortical layer IV of the ventral medial prefrontal cortex. ***C***, Similar results were obtained as shown in ***A***. TBS-induced LTP was nearly completely blocked in slices from neutron-irradiated mice relative to controls. Inset, Representative traces collected during baseline (solid line) and 60 min post-TBS (dotted line). ***D***, Field EPSP slope was significantly reduced 60 min post-TBS in slices from 18 cGy mice compared with controls. *N* = 9 slices per group. ***p < 0.0001 (two-tailed *t* test).

We assessed LTP 6 months following irradiation in acute dorsal hippocampal and ventral medial prefrontal cortex brain slices prepared from neutron-irradiated and concurrent control mice (Fig. 3). The delivery of five theta burst stimulations to control hippocampal slices caused the predicted rapid increase in the slope of the field fEPSPs followed by decay over 10 min to a stable plateau at 155 ± 3.8% [95% CI (147, 164)] above pre-TBS baseline (Fig. 3*A*). A similar rapid increase was observed following TBS in slices from neutron-irradiated mice but the level of potentiation achieved 60 min post-TBS was significantly reduced to 122 ± 3.4% above pre-TBS baseline [95% CI (114, 130), *p* < 0.001; Fig. 3*B*]. Similar results were obtained in TBS-induced LTP in the ventral medial prefrontal cortex slices (Fig. 3*C*). Chronic neutron irradiation caused a nearly complete block in LTP relative to controls 151 ± 2.8% [95% CI (144, 157) and 106 ± 4%, 95% CI (96.6, 115), respectively; *p* < 0.0001; Fig. 3D].

Past studies demonstrate somewhat mixed effects of acute GCR exposures on LTP. Although moderate acute doses of ^56^Fe radiation (200 cGy) disrupt hippocampal LTP (Vlkolinský et al., 2007), some lower doses of acute GCR irradiation (25 cGy, ^28^Si) may conversely enhance LTP (Raber et al., 2014). The LTP detriments we observe after 18 cGy chronic neutron irradiation may suggest that neutron radiation has effects similar to relatively higher doses of single-ion GCR. Nevertheless, the altered network-level hippocampal and cortical LTP we observe suggests that mice chronically exposed to low-dose and dose-rate neutron radiation may display diverse behavioral deficits.

### Neutron irradiation induces deficits in mouse behavior

Animal behavior, ranging from sociability to anxiety, fear, learning, and memory, arises from the intricate interplay between molecular-, cellular-, and network-level mechanisms within the brain (Lynch, 2004; Pape and Pare, 2010; Johansen et al., 2011; Bhattacharya and Klann, 2012; Hulbert and Jiang, 2017). Therefore, we evaluated the impact of chronic, low dose rate, neutron irradiation on a range of core behaviors that would present a risk to astronauts on a deep space mission, using mice from the same cohort used in the neurophysiology studies. Following the social interaction test (SIT), a separate cohort of animals was sequentially administered the following behavioral tasks: NOR, OiP, LDB, FST, and FE memory testing. All behavioral tests were conducted between 12 and 16 weeks following the conclusion of chronic neutron irradiation.

**Figure 4.**
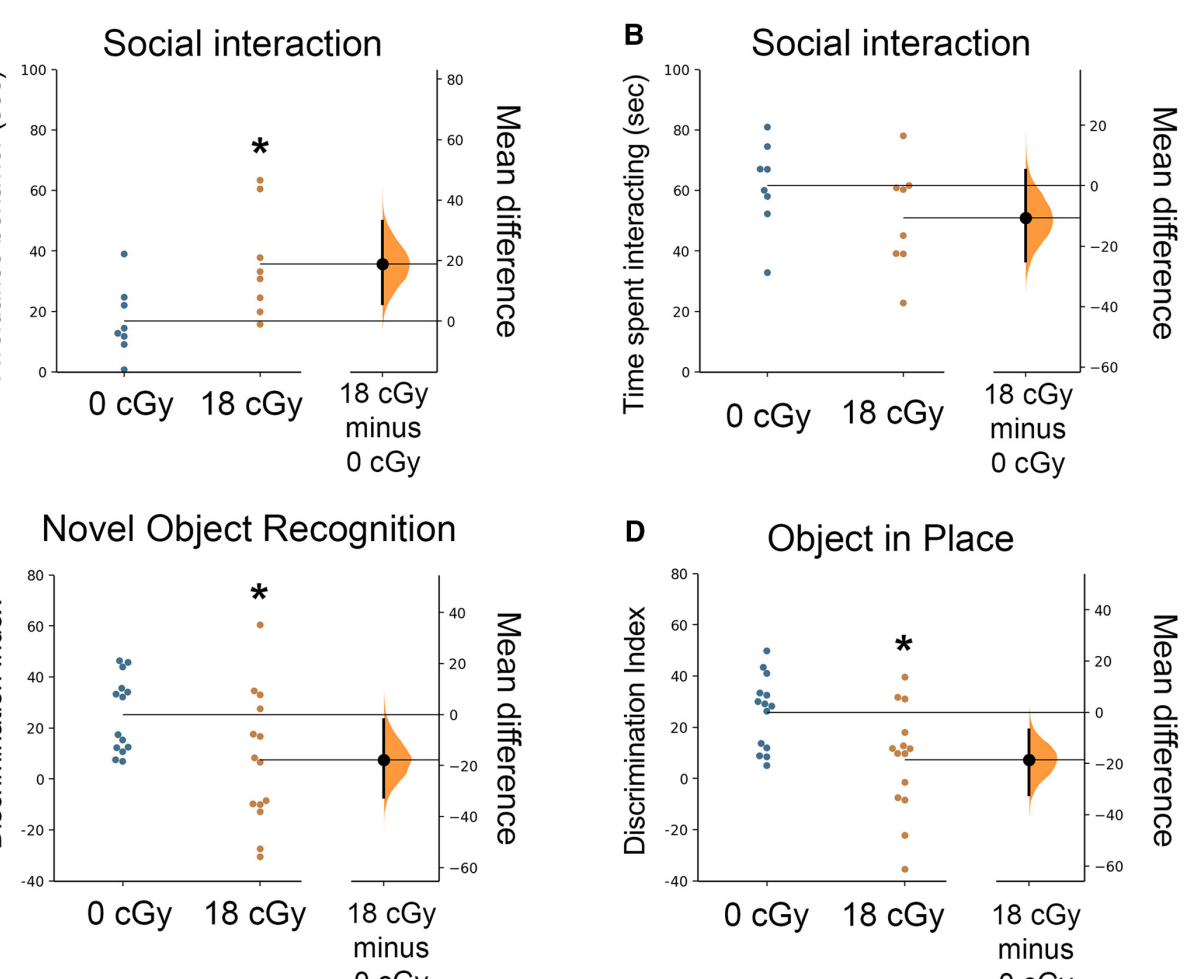
Space-relevant, low dose-rate neutron irradiation disrupts cognition. ***A***, ***B***, Social interaction behavior testing of mice 3 months after the conclusion of neutron irradiation (18 cGy cumulative dose) reveals an increase in avoidance behavior during 10 min trials compared with un-irradiated control mice, whereas the total time spent interacting did not change. ***C***, Disrupted performance on a NOR task by irradiated mice indicates a significant decrement in novelty recognition memory. ***D***, The spatial exploration behavior analyzed for the OiP task demonstrates that neutron irradiation impairs spatial memory retention as manifested in a reduced preference to explore the novel placement of objects. Data are presented as mean ± SEM (***A***, *N* = 8 per group; ***B***–***D***, *N* = 14 per group). *p < 0.05 (Mann–Whitney’s two-tailed, nonparametric *t* test) compared with controls.

Social interaction behaviors are known to be dependent on hippocampal and medial prefrontal cortex circuits (Finlay et al., 2015; Ko, 2017). Mice that had been previously habituated with cage mates were allowed to interact with a novel mouse in a barrier-free arena. Total time the test mouse spent interacting with the novel mouse or actively avoiding social interactions initiated by the novel mouse were recorded following established protocols (Winslow, 2003; Sato et al., 2013b; Gunaydin et al., 2014). Interestingly, neutron-irradiated mice spend a greater amount of time actively avoiding social interactions than do control mice, consistent with perturbed hippocampal and medial prefrontal cortex function [0 cGy: 16.8 ± 4.1 s, 95% CI (7.1, 26.5); 18 cGy: 35.7 ± 6.3 s, 95% CI (20.9, 50.5); *p* = 0.028; Fig. 4*A*; Winslow, 2003; Sato et al., 2013b; Gunaydin et al., 2014]. However, we find that control and neutron-irradiated mice spend a similar amount of time socially interacting with a novel mouse [0 cGy: 61.6 ± 5.2 s, 95% CI (49.2, 73.9); 18 cGy: 50.9 ± 6.2 s, 95% CI (36.2, 65.5); *p* = 0.33; Fig. 4*B*].

Like social interactions, recognition memory tasks are known to require proper hippocampal and medial prefrontal cortex signaling (Finlay et al., 2015; Ko, 2017). To further assess the functional impact of low dose-rate neutron exposure on episodic and spatial memory function, we performed two distinct behavioral tasks: NOR and OiP. For both tasks, the total combined exploration times of the familiar and novel objects were comparable between treatments (data not shown), suggesting no confounding alterations in locomotion or exploratory behavior. Analysis of novel object preference in the NOR test, as indicated by the discrimination index, indicates that neutron irradiation impairs novel object recognition memory relative to control mice [0 cGy: 25.3 ± 4.0 s, 95% CI (16.7, 33.8); 18 cGy: 7.5 ± 6.9 s, 95% CI (−7.4, 22.5); *p* = 0.044; Fig. 4*C*]. Furthermore, neutron-irradiated mice show similarly reduced discrimination of objects moved to a novel location in the OiP task compared with control animals [0 cGy: 25.8 ± 3.8 s, 95% CI (17.7, 34.0); 18 cGy: 7.2 ± 5.6 s, 95% CI (−4.8, 19.2); *p* = 0.027; Fig. 4*D*]. Performance on the NOR and OiP tasks both require intact processing within and communication among the hippocampus, medial prefrontal cortex, and perirhinal cortex, albeit potentially in varying degrees (Squire et al., 2004; Warburton and Brown, 2015). Thus, deficits in the NOR and OiP memory tasks demonstrate the widespread detrimental impacts of protracted, low dose-rate neutron exposures on the CNS.

**Figure 5.**
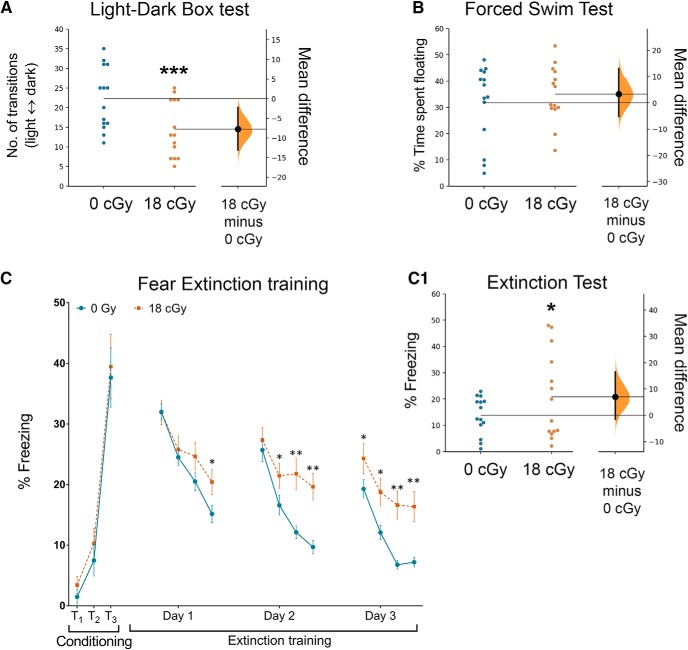
Space-relevant, low dose-rate neutron irradiation elicits anxiety-like behavior. ***A***, Neutron irradiation increases anxiety-like behavior as the irradiated mice exhibit reduced numbers of transitions between the light and dark chambers in the LDB test. ***B***, Irradiated mice did not show depression-like behavior on the FST. ***C***, Last, neutron irradiation compromised fear extinction memory function. Mice showed elevated freezing following a series of three tone–shock pairings (0.6 mA, T_1_–T_3_). Subsequently, 24 h later, fear extinction training was administered every 24 h (20 tones) for 3 d. All mice showed a gradual decrease in the freezing behavior (Days 1–3), however, irradiated mice spent a significantly higher time in freezing compared with controls. Twenty-four hours after extinction training, control mice showed abolished fear memory compared with neutron-exposed mice (***C1***, inset). Data are presented as mean ± SEM (***A***–***C***, ***C1***, *N* = 14 per group). *p < 0.05, **p < 0.01, ***p < 0.001 (Mann–Whitney’s two-tailed, nonparametric *t* test) compared with controls.

Radiation exposures are often observed to alter mood (Butler et al., 2006; Tang et al., 2012; Caceres et al., 2013; Yen et al., 2014), with low doses of acute charged particle irradiation producing persistent increases in anxiety- and depression-like behavior in mice (Parihar et al., 2016, 2018). To determine whether chronic exposures to low dose-rate neutrons also trigger anxiety- and depression-like behavior, mice were administered the LDB test and the FST. The LDB test contrasts the natural inclination of mice to explore novel environments with their aversion to brightly lit spaces, thus evaluating increased anxiety based on greater preference for the dark versus light compartments of the testing arena (Bourin and Hascoët, 2003). Compared with un-irradiated controls, neutron-exposed mice show a reduction in the number of light–dark transitions [0 cGy: 22.3 ± 2.1 transitions, 95% CI (17.7, 26.8); 18 cGy 14.5 ± 1.9 transitions, 95% CI (10.4, 18.6); *p* = 0.009; Fig. 5*A*]. In addition, the neutron-irradiated animals spent a significantly reduced percentage time in the light compartment [0 cGy: 80.9 ± 2.5, 95% CI (76.6, 85.9); 18 cGy: 52.5 ± 3.3, 95% CI (45.9, 58.3); *p* < 0.0001]. The amygdala is understood to be an important regulator of anxiety behavior (Tye et al., 2011; Calhoon and Tye, 2015), suggesting an additional brain structure that is disrupted by neutron irradiation. Conversely, chronic neutron irradiation does not alter the time mice spend floating in the FST, indicating no effect of low-dose and dose-rate radiation on depression-like behavior [0 cGy: 31.7 ± 3.9 s, 95% CI (28.8, 41.3); 18 cGy: 35.0 ± 2.9 s, 95% CI (23.2, 40.2); *p* = 0.92; Fig. 5*B*].

Finally, we examined how the extinction of fear memory, the active process of dissociating learned responses to prior adverse events (Cain et al., 2003), is altered by chronic neutron irradiation. In the conditioning phase, control and neutron-exposed mice were administered to three tone–shock pairings (T_1_–T_3_; 120 s, 16 kHz tone followed by a 1 s, 0.6 mA shock; 2 min interval) and display a comparable percentage of time spent freezing during fear acquisition [T_1_: 1.48 ± 1.15%, 95% CI (−1.01, 3.97) and 3.42 ± 1.43%, 95% CI (0.33, 6.51), *p* > 0.10; T_2_: 7.49 ± 2.60%, 95% CI (1.87, 13.11) and 10.31 ± 2.49%, 95% CI (4.92, 15.69), *p* > 0.10; T_3_: 37.67 ± 4.88%, 95% CI (27.12, 48.22) and 39.48 ± 5.32%, 95% CI (27.99, 50.97), *p* > 0.1; *N* = 14 each group, 0 and 18 cGy, respectively; Fig. 5*C*]. During subsequent fear extinction trials in a new context (20 tones/d, 2 min interval, no shock), control mice exhibit a gradual decrease in freezing behavior over the course of the trial (Fig. 5*C*, Days 1–3; data show average response per 5 tones). 95% CIs for the last five tones for each extinction training day were as follows: Day–1 (12.32, 18.01) and (16.3, 24.59), Day–2 (7.44, 11.95) and (15.25, 24.02); Day–3 (5.59, 8.80) and (11.38, 21.36); 0 and 18 cGy, respectively. Importantly, we find a significant radiation effect across extinction trials (*F*_(1,1734)_ =29.57, *p* < 0.0001, two-way RM ANOVA). On the final day of extinction testing only three tones were administered (2 min interval; Fig. 5*C1*). Control mice show more complete fear extinction than neutron-irradiated animals [0 cGy: 13.8 ± 1.7% freezing, 95% CI (9.66, 17.97); 18 cGy: 20.8 ± 2.7% freezing, 95% CI (11.32, 30.27); *p* = 0.04]. Impaired fear extinction is considered to represent a post-traumatic stress disorder-like phenotype (Chang et al., 2009; VanElzakker et al., 2014) and indicates a diffuse dysfunction of neural processes involving the hippocampus, medial prefrontal cortex, and amygdala (Quirk and Mueller, 2008; Chang et al., 2009).

Importantly, cohort averaged data does not provide an indication of how frequently or how severely neurocognitive performance is impacted. Therefore, we further analyzed individual animal performance data using KDE to generate a performance probability profile (Jewell et al., 2018). The generated performance probability profiles were then used to determine the percentage of neutron-irradiated mice that exhibit significantly altered behavior, defined as performance in a task ≤5th percentile of the performance metric observed for the concurrent control mice. Relative to our 5% impairment threshold in control mice, significantly more neutron-irradiated mice show decrements on the NOR, OiP, LDB, and FE tests (25–41%, *p* < 0.0001, Fisher’s exact test; Table 3). On the SIT test, 15% of irradiated mice exhibited decreased time interacting with the novel mouse and 34% of the irradiated cohort exhibited significantly increased time spent in avoidance behavior (*p* = 0.019 and *p* < 0.0001, respectively). The data from the KDE analysis was then substituted into a numbers needed to harm (NNH) algorithm (Jewell et al., 2018). Extrapolating from our NNH analysis, we predict that 1 in every 5.1 astronauts would experience increased anxiety-like behavior during a mission to Mars, whereas 1 in every 2.8 astronauts would experience difficulty with episodic memory.

**Table 3.** Kernel density estimation for neutron radiation-induced behavioral deficits

**Test**	**Endpoint**	**TD concept**	**TD**	**Sham, %**	**Neutron, %**	***p***	**ARR**	**NNH**	**CI**
**SIT**	Interact time	Bottom %	>42.1	5.0	34.4	0.019	29.3	3.4	2.5,5.2
**NOR**	DI	Bottom %	<0.85	5.0	41.1	<0.0001	36.0	2.8	2.1,3.9
**OiP**	DI	Bottom %	<1.0	5.0	34.6	<0.0001	29.6	3.4	2.5,5.2
**LDB**	No. transitions	Bottom %	<8.0	5.0	24.8	0.0001	19.8	5.1	3.4,9.7
**FST**	Time floating, %	Upper %	>55.7	5.0	0.7	NS	0.7	143	14.4, infinity
**FE**	Time freezing, %	Upper %	>26.2	5.0	36.8	<0.0001	31.8	3.1	2.4,4.7

DI, Discrimination index; TD, threshold dose; ARR, absolute relative risk; NS, not significant.

Fisher’s exact *t* test, two-sided, α < 0.05.

## Discussion

Currently, NASA, other governments and multiple commercial enterprises are racing to develop the capabilities necessary to send humans to Mars by the 2030 s. However, a number of health concerns related to prolonged radiation exposures in space have compelled increased research into how ionizing radiation affects the brain. Past findings abundantly documenting behavioral impairments arising in multiple rodent models of acute cosmic radiation exposures (Rabin et al., 2011; Cherry et al., 2012; Haley et al., 2013; Davis et al., 2015; Parihar et al., 2015b; Whoolery et al., 2017; Carr et al., 2018; Jewell et al., 2018), have inspired the utilization of new experimental paradigms to even more realistically simulate the space radiation environment. Here, we describe the first study assessing the consequences of chronic, low dose and low dose rate, exposure to high LET charge particle recoils from neutron interactions and accompanying low LET photons on CNS functionality. Importantly, our study specifically matches anticipated dose rates relevant to upcoming human space exploration (Zeitlin et al., 2013). Despite the differences between protracted neutron exposures on Earth and GCR exposures in space, our experimental protocol reasonably simulates exposures from GCR during prolonged mission in deep space.

Therefore, we have endeavored to examine the risks posed by chronic, low-dose and dose-rate exposures to high LET radiation across levels of CNS function, spanning from cellular- to whole animal-level impacts. Fundamentally, disruptions of animal behavior or neural network function arise from underlying disturbances in the activity of individual neurons. The reduced intrinsic excitability of CA1 pyramidal neurons we observe following chronic neutron irradiation is entirely consistent with our previous findings that acute charged particle exposures hyperpolarize neuronal RMPs within the hippocampus (Sokolova et al., 2015) and perirhinal cortex (Parihar et al., 2018). We do not probe the molecular underpinning of suppressed neuron excitability in the current study, yet low-dose (10 cGy) ionizing radiation is sufficient to downregulate neuronal expression of ion channels (Lowe et al., 2009; Lowe and Wyrobek, 2012). Additionally, reactive oxygen species generated by charged particle radiation exposure (Suman et al., 2013) can directly alter the activity of ion channels found on neurons (Choi and Lipton, 2000; Todorovic and Jevtovic-Todorovic, 2014), potentially underlying the effects we observed.

In agreement with the finding that chronic irradiation reduces the excitability of hippocampal neurons, the frequency of excitatory synaptic inputs to CA1 pyramidal neurons are also suppressed. Indeed, reduced excitatory synaptic tone may be correlated with our observed changes in intrinsic properties. Acute GCR irradiation similarly lessens excitatory synaptic inputs within the hippocampus (Sokolova et al., 2015) and perirhinal cortex (Sokolova et al., 2015). Furthermore, acute GCR exposures reduce excitatory neurotransmitter expression (Machida et al., 2010; Carr et al., 2018) and alter the morphology of dendritic spines on hippocampal and cortical neurons (Parihar et al., 2015b, 2016; Carr et al., 2018). Such changes are entirely consistent with our observation of reduced excitatory signaling among neutron irradiated hippocampal neurons. Moreover, acute proton irradiation enhances GABA release by hippocampal interneurons (Lee et al., 2017), which may also contribute to our noted suppression of network activity.

Such disruptions of excitatory synaptic signaling to CA1 pyramidal neurons are implicated in reducing theta frequency (3–10 Hz) oscillations within the hippocampus and thereby weakening the capacity for LTP (Buzsaki, 2002; Sokolova et al., 2015). Thus, the LTP deficits we observe within hippocampal and medial prefrontal cortex neuronal networks are consistent with our single-cell recordings. Interestingly, acute radiation exposure at low doses appears to enhance hippocampal LTP (Raber et al., 2014) and molecular pathways associated with synaptic plasticity (Lowe et al., 2009). However, compensatory mechanisms that can effectively respond to acute irradiation may become overwhelmed by the chronic radiation exposures that astronauts will face during prolonged spaceflights.

Altogether, we have not only identified behavioral deficits associated with the cellular- and circuit-level alterations in hippocampal and medial prefrontal cortex function, but also what appears to be generalized disruptions of CNS function by chronic neutron irradiation. Our testing represents a realistic model of the risks associated with the low-dose and low dose-rate radiation astronauts will experience over the course of a deep space mission. Although our electrophysiological findings provide compelling evidence that chronic, low-dose and dose-rate irradiation perturbs hippocampal and medial prefrontal cortex activity, our behavioral testing suggests that functional deficits additionally extend to the perirhinal cortex, amygdala, and possibly beyond. The spectrum of behavioral deficits we observe in social avoidance, anxiety, impaired FE memory, and difficulties recognizing location and object novelty, would clearly impair the abilities of astronauts needing to respond quickly, appropriately, and efficiently to unexpected situations that arise over the course of a mission to Mars. Moreover, past studies have found an inverse relationship between increased animal anxiety and decreased depression-like behavior in the FST (Estanislau et al., 2011) that could account for our observed result. The anxiety-like behavior of neutron-irradiated mice implicates additional brain regions that appear to be disrupted by chronic, low dose-rate exposures and suggests that astronauts will also be at risk for developing mood disorders during long duration space flights. Importantly, our findings mirror those obtained with multiple charged particle types, in which similarly low doses were instead administered over acute (minutes) times (Rabin et al., 2011; Cherry et al., 2012; Haley et al., 2013; Davis et al., 2015; Parihar et al., 2015b, 2016, 2018; Whoolery et al., 2017; Carr et al., 2018; Jewell et al., 2018). The fact that similar and significant behavioral deficits arise following markedly different dose rate irradiations argues in favor of unique CNS responses to cosmic radiation exposure, distinct from other organ systems, and one that poses a potent hazard to all levels of the nervous system.

However, precisely translating our findings into tangible risk estimates for such a dynamic and complex system as the brain remains a challenge. Such a task is also distinctly different from studies estimating radiogenic cancer risk where a wealth of epidemiological data exists. The KDE analysis we perform is a useful tool to assign a level of behavioral performance that can be considered to represent a severe compromise of cognitive function (Table 3). Additionally, we can use the KDE metric to quantify the numbers of individuals within the test population that display a severe impairment (Jewell et al., 2018). For the purposes of this study, the level of performance within a given test that was considered to represent severe impairment was set at the 5th percentile of the control population. Substituting the derived values for severely impaired individuals into the NNH algorithm then provides estimates of absolute risk and the frequency that severely impaired performance in a given outcome measure will occur following chronic neutron irradiation. Shockingly, for multiple behavioral measures, we calculate an NNH of ∼3.5–5. Thus, in a crew of five astronauts traveling to Mars, we would expect at least one member to display severe deficits in each of those cognitive functions by the time they return to Earth.

Until now, it was unclear how the CNS might respond to chronic low dose exposures simulating the space radiation environment. Low dose rates, like the ones used in our current study, produce infrequent particle traversals across non-proliferative neurons, including across very small targets such as dendritic spines and synapses (Cacao et al., 2018). Thus the evoked damage may be insufficient to trigger an adaptive or compensatory response in the brain. Therefore, the sensitivity of the CNS to space radiation may instead reflect the cumulative effect of an absorbed dose over time that perturbs the delicately balanced circuit connectivity and coordinated responses essential to proper CNS function. Protracted exposures may further augment adverse effects, as plasticity becomes compromised and the rewiring of damaged circuits becomes imperfect in the context of chronic irradiation. As NASA continues to develop the capability to produce increasingly realistic chronic GCR exposures, we will be able to even more accurately evaluate the consequences of low dose-rate exposures on CNS function (Chancellor et al., 2018).

Here we report the first measurements of functional outcomes in the rodent brain following space-relevant, low-dose and low dose-rate exposure to neutrons. These cosmic radiation simulations reveal marked disruptions in cellular- and network-level electrophysiological function and whole-animal alterations in behavioral performance. Collectively, our data clearly demonstrate that radiation-induced damage to the CNS accumulated over the course of deep space travel poses considerable risks for astronaut performance and health. Confounding efforts to elucidate the mechanistic underpinnings of our identified disruptions is the lack of a consensus on the radiosensitive target in the CNS. Whereas in other organ systems, radiation effects are largely attributable to DNA damage and increased cell death, such effects are not likely to play a significant role in the CNS. Although our current study does not address the molecular changes underlying the deficits we report, it does present a framework for CNS mechanism to target in future studies. In the long-term, the nature of the radiation environment in space will not deter our efforts to travel to Mars, but it may be the single biggest obstacle humankind must resolve to travel beyond the Earth’s orbit.

## Synthesis

Reviewing Editor: Liset Menendez de la Prida, Instituto Cajal CSIC Decisions are customarily a result of the Reviewing Editor and the peer reviewers coming together and discussing their recommendations until a consensus is reached. When revisions are invited, a fact-based synthesis statement explaining their decision and outlining what is needed to prepare a revision will be listed below. The following reviewer(s) agreed to reveal their identity: Niraj Desai.

Your ms was reviewed by two independent neuroscientists and myself. We also consulted with a NASA expert. During the consultation there was some discussion regarding some major methodological issues and reviewers disagree on whether we should reject or not the paper at this point given the need for substantial revision requiring more than two moths.

However, we all agree your data is unique and that we should let you the chance to consider all these concerns. Thus, we decided to give you the chance of resubmitting a revised version. I should stress however, the need for a major and thorough revision. Your paper will be reviewed again and I will then look for a consensus recommendation.

Here is a synthesis of the reviewers' assessment. Given the relevance, I copy the NASA expert's comments at the end.

Major comments:

1- Potential confounding effects of radiation not accounted for (visual impairment and other physiological effects) to explain behavioural effects (see Ramos et al 2019 Experimental Eye Research; doi.org/10.1016/j.exer.2019.01.019). Several behavioral tasks in which radiation leads to functional deficits seem to involve visual discrimination. PLease, address this properly.

2- Additional confirmatory analysis are required to validate changes of intrinsic excitability and synaptic responses. First, change in resting potential seems a bit odd because there are no (significant) changes in input resistance or sag, which suggests there were no changes in leak, H, or GIRK currents. We are thus unclear on what may produce the resting potential change? (The rheobase effect could simply reflect the resting potential one.) The authors might address this question by analyzing intrinsic excitability more thoroughly than they do - for example, by measuring f-I curves, threshold potential, spike height, afterhyperpolarization, spike frequency adaptation. The current steps ranged between -200 pA and +750 pA, and yet the manuscript reports rather little about the responses to them. Second, intrinsic excitability measurements were done without blocking synaptic currents. This is not ideal - because, for example, a hyperpolarized resting potential could be the result of a decrease in excitatory background rather than a change in intrinsic channels. The confound is unlikely to be a major one in this case because the synaptic background (Fig. 1F) looks rather low. Even so, the authors should demonstrate that this is true for their recording conditions by recording intrinsic excitability before and after washing in synaptic blockers. This could be done simply with tissue from control animals.

3- Pseudoreplication issues. Patch clamp data of Figure 1 are derived, for each group, from 27 recordings taken from 7-8 animals. Each recording is treated as an independent observation in the analysis, but this treatment might not be valid. Recordings from a given animal might be correlated with other recordings from that same animal. The issue, called “pseudoreplication” (Lazic, BMC Neuroscience, 2010), has received a lot of attention in neuroscience recently. The authors should try to reanalyze those data “by animal” rather than “by recording.”

4- Additional analysis of behavioral experiments. The battery of behavioral tests is mostly well explained (if only by referring to conventional methods, previously described). One test on which I would like more detail is freezing. I am familiar with FreezeFrame. Its “automated” software includes some choices about how to block time and set a freezing criterion. What choices did the authors make?

5- Concerns regarding sEPSC decay. Whole cell voltage clamp on neurons with dendrites can be tricky, especially when one is trying to characterize a current from a distal site. What criteria did the authors use to determine that the synaptic currents they measured were well clamped? A rise time cutoff? A threshold? A stringent series resistance criterion and/or steps to minimize series resistance filtering? One thing I would recommend is to measure charge (by integrating each current). Charge is less sensitive to the effects of cable filtering.

6- The ms lacks many methodological details and should be thoroughly revised. Here is a list of concerns:

a) What series resistance, resting potential, and input resistance cutoffs did they use to determine whether a given recording should be included in their data set?

b) Did they balance the bridge and use pipette capacitance compensation when recording in current clamp?

c) What holding potential did they use in voltage clamp?

d) The authors are confident that the EPSCs are indeed excitatory because why? The GABA reversal was negative relative to holding? e) In the mPFC LTP experiments, did they do anything to block inhibition? In neocortex, that tends to be a big problem when stimulating extracellularly.

f) The extrapolation to NNH is not sufficiently justified and the cited paper does not include estimation of NNH

g) There is an abusse of abbreviation of brain regions and behavioral tests. Expanding these in the results would improve readability.

Some minor comments:

7- In one or two places, the authors report results to an unneecesary level of precision (control animals spent “80.899 +/- 2.465” percent of their time in the light area). In the text, the freezing responses are referred to as pure numbers rather than as percentages.

8- In figure 1 use sEPSC instead of EPSC for clarity

9- 449-453 and 475-483 are more appropriate in the discussion.

10- Figures 3A and 3B are incorrectly referenced in the results.

11- Relating the findings to radiation exposure during cancer radiotherapy could expand the relevance of the study

Finally. Here are commenst by the NASA expert. Please, address all of them carefully.

- Title should have neutrons in it - to differentiate from more harmful GCR particles (Galactic cosmic rays).

- Throughout the manuscript (significance etc) they talk about space radiation - but I feel that they need to be very specific -

- The real issue is likely due to secondary neutrons that interact with other particles - such as the space craft etc. Which they acknowledge - it is not clear if they are trying to model ambient neutrons in space or those from interactions with shielding (which are likely more important).

- I could not find much info on the current neutron rate/dose in space (neutrons decay in 10min) but in the Nelson 2016 paper they posit about 20-30mGy dose over three years.

- Paper is important as it is one of the first to do long-term exposures.

- The KDE is a smoothing function (estimate probability distribution of a random variable) and based on the results don't tell us how they fit the data - they simply grab the top 95% - but sort of poor rationale at least for me - They make some strong assertions based on the KDE - so they need to model this a bit better - details like the bandwidth that they used... there is an optimal (ie silvermans ) choice. KDE is not good for uniformally distributed data either.

- Ephsyio data looks good, but in some instances the resultant significance appears comes to come from a few animals in each that seem to drive the significance.

Overall interesting and important for space travel, but again neutrons are not overly important relative to heavy ions; I think if you decide to keep this manuscript, I would force them to be very specific on which types of neutrons they are modeling and that it is distinct from other GCR particles.

## Author Response

We appreciate the opportunity to respond to the critiques, and we have also taken the liberty to include 95% confidence intervals for all our data sets in the main manuscript, thereby increasing the statistical visibility of our data sets. Below is a point-by-point response to the reviewers' comments:

Major comments:

1- Potential confounding effects of radiation not accounted for (visual impairment and other physiological effects) to explain behavioral effects (see Ramos et al 2019 Experimental Eye Research; doi.org/10.1016/j.exer.2019.01.019). Several behavioral tasks in which radiation leads to functional deficits seem to involve visual discrimination. Please, address this properly.

This is a good point but under the current context of our studies is simply not a concern. The reference quoted refers to the control of ocular tumor in the clinic through brachytherapy techniques or external beam radiation. The most common form of radiation therapy to treat ocular tumors is brachytherapy which brings the radiation source close to the cancerous tissue. Routine absorbed doses to the eye range from 70 -100 Gy and can lead to vision impairments within 6 months to 3 years following treatment. The critical feature here is that the radiation dose is 400 times higher than what was used in this study and our animals exhibited no signs of ocular complications whatsoever. Furthermore, in our behavioral tests, control and irradiated animals exhibited similar exploration to the objects during spontaneous exploration tasks indicating functional vision. For example, total exploration for both objects on the NOR task was 41{plus minus}3.8s (control) and 40{plus minus}2.6s (irradiated) and for the OiP task it was 80{plus minus}6.9s (control) and 71{plus minus}5.6s (irradiated). Neither was statistically different.

2- Additional confirmatory analysis are required to validate changes of intrinsic excitability and synaptic responses. First, change in resting potential seems a bit odd because there are no (significant) changes in input resistance or sag, which suggests there were no changes in leak, H, or GIRK currents. We are thus unclear on what may produce the resting potential change? (The rheobase effect could simply reflect the resting potential one.) The authors might address this question by analyzing intrinsic excitability more thoroughly than they do - for example, by measuring f-I curves, threshold potential, spike height, after hyperpolarization, spike frequency adaptation. The current steps ranged between -200 pA and +750 pA, and yet the manuscript reports rather little about the responses to them. Second, intrinsic excitability measurements were done without blocking synaptic currents. This is not ideal - because, for example, a hyperpolarized resting potential could be the result of a decrease in excitatory background rather than a change in intrinsic channels. The confound is unlikely to be a major one in this case because the synaptic background (Fig. 1F) looks rather low. Even so, the authors should demonstrate that this is true for their recording conditions by recording intrinsic excitability before and after washing in synaptic blockers. This could be done simply with tissue from control animals.

We agree that important details of intrinsic responses were previously excluded and we have now expanded the analysis we include. Based on the estimation statistics we have added to the manuscript, the 12.0 MΩ decrease in input resistance of irradiated mice qualifies as a small effect based on a Cohen's d of -0.43. However, we acknowledge that this result is not significant based on traditional statistical methods. Determining how to deal with such discrepancies will be important as neuroscientists seek to move beyond statistics relying on the binary verdicts of P-values.

With the F-I curve we now include, we show that neutron-irradiated neurons are less excitable across the range of current injections we performed. In a further analysis of the F-I responses performed only in a subset of neurons with equivalent resting membrane potentials, we still observe attenuated responses in irradiated neurons. However, we believe that the full dataset better represents the underlying biological finding that neutron irradiation decreases the excitability of CA1 pyramidal neurons. We have also added additional analysis of the AP thresholds, heights, widths and afterhyperpolarization, although we observe no significant changes in any of these measures (although the Cohen's d for the decrease in afterhyperpolarization following irradiation indicates a small effect).

We appreciate your suggestion of recording intrinsic excitability in the presence of synaptic signaling blockers to preclude any influence of synaptic currents on the intrinsic properties. However, as you point out, background synaptic activity appears to be relatively sparse in both of our treatment groups, making it unlikely to exert much of an impact on the intrinsic properties we measured. The control mice in the current experiments spent six months housed at Colorado State University under the equivalent conditions to the neutron irradiated mice before being shipped to Stanford University and aged an additional six months. Due to the complications of producing a comparable set of control mice for experiments looking into the impact of adding synaptic blockers on synaptic activity, we were unable to perform additional experiments. Nevertheless, we now note in our manuscript that decreases in the sEPSC frequency of neutron-irradiated mice could be correlated with the hyperpolarized resting membrane potential we observed.

3- Pseudoreplication issues. Patch clamp data of Figure 1 are derived, for each group, from 27 recordings taken from 7-8 animals. Each recording is treated as an independent observation in the analysis, but this treatment might not be valid. Recordings from a given animal might be correlated with other recordings from that same animal. The issue, called “pseudoreplication” (Lazic, BMC Neuroscience, 2010), has received a lot of attention in neuroscience recently. The authors should try to reanalyze those data “by animal” rather than “by recording.”

As you state above, the issue of pseudoreplication, or nested datasets, is definitely gaining attention among electrophysiologists. However, many in the field remain uncertain about how to update their statistical best practices while accounting for the technical constraints of performing slice electrophysiology experiments.

One emerging solution is to analyze datasets using mixed linear model regression analysis (also referred to as multilevel model analysis), where both the degree of within cluster and between cluster variability is accounted for (see Aarts et al., Nat Neurosci, 2014). We have now updated all significance testing between treatment groups in our whole cell electrophysiology experiments to a mixed linear model regression analysis. Furthermore, in line with emerging statistical guidelines at eNeuro, we have additionally included an estimation statistics-based calculation of the effect sizes observed between our treatment groups. We believe that through these two measures we address the concerns related to pseudoreplication without losing the transparency associated with presenting the responses of individual neurons.

4- Additional analysis of behavioral experiments. The battery of behavioral tests is mostly well explained (if only by referring to conventional methods, previously described). One test on which I would like more detail is freezing. I am familiar with FreezeFrame. Its “automated” software includes some choices about how to block time and set a freezing criterion. What choices did the authors make?

The trial viewer function in the FreezeFrame 4 software was used to determine the percentage-based freezing of animals. An investigator blinded to the experimental groups set the motion index threshold representing immobility for each animals individually, based on identifying a trough separating peaks of low values during immobility and higher values associated with motion. The freeze threshold was set between 10 to 16 (0 cGy: 10.51{plus minus}1.86, 18 cGy: 16.1{plus minus}0.94) for these experiments. Freezing behavior was defined as continuous bouts of 1 second or more of immobility. The percentage of time spent freezing during the tone was then calculated for the conditioning, extinction training and the extinction test phases.

5- Concerns regarding sEPSC decay. Whole cell voltage clamp on neurons with dendrites can be tricky, especially when one is trying to characterize a current from a distal site. What criteria did the authors use to determine that the synaptic currents they measured were well clamped? A rise time cutoff? A threshold? A stringent series resistance criterion and/or steps to minimize series resistance filtering? One thing I would recommend is to measure charge (by integrating each current). Charge is less sensitive to the effects of cable filtering.

We have further clarified the criteria used to characterize synaptic currents. sEPSCs detected in Clampfit were screened to only include events with a rise time of <7.5 ms, a peak amplitude of >3 pA, a charge transfer of >25 pC and recordings were excluded where the series resistance increased by >20% of baseline. As suggested, we have replaced our calculation of sEPSC decay times with a measure of the charge transfer during each sEPSC.

6- The ms lacks many methodological details and should be thoroughly revised. Here is a list of concerns:

a) What series resistance, resting potential, and input resistance cutoffs did they use to determine whether a given recording should be included in their data set?

Thank you for pointing out this oversight. We have now included information on these exclusion parameters in our methods section.

b) Did they balance the bridge and use pipette capacitance compensation when recording in current clamp?

Pipette capacitance compensation was performed for all recordings. However, we did not balance the bridge in the current set of experiments. The majority of electrodes used had a low resistance of 2.5-3.5 MΩ (with an overall range of 2.5-5 MΩ), thereby minimizing any introduced error in recorded voltages. Under the recording conditions utilized in these experiments we have noticed only minimal differences between recordings when we did or did not include a bridge balance adjustment. Furthermore, having an unbalanced bridge has a limited effect on the current injections received by recorded neuron, thus it has minimal impact on either the rheobase current of F-I changes we observe. We do recognize that it is important to consider the potential errors that could be introduced by an unbalanced bridge in recordings with elevated electrode resistances.

c) What holding potential did they use in voltage clamp?

The holding potential of -65 mV during voltage clamp recordings has been further clarified in the methods section and added to the results section (and the corresponding figure legend).

d) The authors are confident that the EPSCs are indeed excitatory because why? The GABA reversal was negative relative to holding?

To measure putative EPSCs, neurons were voltage clamped at -65 mV, which is depolarized relative to the expected EGABA of CA1 pyramidal neurons (~-71 mV, see Dargaei et al., PNAS, 2018). During analysis, only inward currents were analyzed as sEPSCs. However, measuring the reversal potential of likely sEPSCs was beyond the scope of the current experiments, so we are unable to comment on whether EEPSC did indeed surpass the action potential threshold of the recorded neurons. Nevertheless, we believe it is reasonable to label these inward currents as sEPSCs.

e) In the mPFC LTP experiments, did they do anything to block inhibition? In neocortex, that tends to be a big problem when stimulating extracellularly.

Response: Picrotoxin (GABAA receptor blocker) was not included in the bath solution. On page 9, second paragraph within the Methods section of the paper we describe the care taken in positioning the electrodes within the mPFC and include a reference by Kirkwood and Bear, 1994, which demonstrates that theta burst-induced LTP can be achieved without the use of GABAA receptor blocker if the recording and stimulating electrodes are positioned in cortical layers III and IV, respectively.

f) The extrapolation to NNH is not sufficiently justified and the cited paper does not include estimation of NNH.

We thank the reviewer for pointing out the fact that the NNH was indeed not cited in the Jewell paper. We have substituted the details of the website-based algorithm we used to calculate the NNH. http://araw.mede.uic.edu/cgi-bin/nntcalc.pl.

We are unclear why the reviewer thinks that the extrapolation to NNH was not sufficiently justified. The journal BMJ Best practice recommends such an approach for clinical trials to either calculate Numbers Need to Harm or Numbers Needed to Treat. The NNH is an important measure in evidence-based medicine and helps physicians decide whether it is prudent to proceed with a particular treatment which may expose the patient to harm while providing therapeutic benefits. Internationally renowned medical journals routinely publish articles related to clinical neuroscience that use NNH (e.g., DOI:https://doi.org/10.1016/S0140-6736(15)60715-2). Thus the validity of the technique is hard to dispute. This type of analysis has not been widely used in rodent studies, and while it is unusual, the design of our study, makes the application of this type of risk analysis appropriate.

g) There is an abuse of abbreviation of brain regions and behavioral tests. Expanding these in the results would improve readability.

We appreciate this concern and have expanded the abbreviations for the brain regions (mPFC, PRC). We have tried to minimize our use of abbreviations for the various cognitive tasks.

Some minor comments:

7- In one or two places, the authors report results to an unnecessary level of precision (control animals spent “80.899 +/- 2.465” percent of their time in the light area). In the text, the freezing responses are referred to as pure numbers rather than as percentages.

This has been fixed

8- In figure 1 use sEPSC instead of EPSC for clarity

Agreed, this change has been made.

9- 449-453 and 475-483 are more appropriate in the discussion.

These sections have been removed and incorporated into the discussion at your request (refer to pgs. 29-30).

10- Figures 3A and 3B are incorrectly referenced in the results.

Thank you - this has been fixed

11- Relating the findings to radiation exposure during cancer radiotherapy could expand the relevance of the study.

While we acknowledge this idea, we have specifically tried to avoid this since the radiation paradigm used here is distinctly different than any current radiotherapeutic protocol. The low dose rate of neutrons used here are not practical for the control of cancer growth and are not used in the clinic. Higher dose rate neutron sources are used in clinical practice but represent very different irradiation parameters from those described in this manuscript. As a consequence, we prefer to avoid any such clinical comparisons that could be mistakenly construed and lead to some unnecessary criticism from radiation oncologists.

Finally. Here are comments by the NASA expert. Please, address all of them carefully.

Title should have neutrons in it - to differentiate from more harmful GCR particles (Galactic cosmic rays).

Agreed

Throughout the manuscript (significance etc) they talk about space radiation - but I feel that they need to be very specific.

The manuscript has been checked carefully and is accurate regarding our usage of space radiation.

The real issue is likely due to secondary neutrons that interact with other particles - such as the space craft etc. Which they acknowledge - it is not clear if they are trying to model ambient neutrons in space or those from interactions with shielding (which are likely more important).

There can be no doubt that neutrons arising from GCR interactions with the spacecraft, body and planetary surface will contribute to the total dose that astronauts receive (see below). There are also other terrestrial based neutron sources that can deliver higher doses and dose rates of neutrons - typically used in the clinic for the control of malignancies. Importantly, our studies were not designed to model neutron exposures in space, but rather the low dose rates of ionizing radiation found in space. At present this can only be accomplished at the CSU facility implementing 252Cf fission neutrons, which deliver very low dose rates (1mGy/day) of neutrons and gamma rays, as stated in this manuscript. As a point of interest, the energy spectra of the fission neutrons are similar to those within the spacecraft after GCR interactions with 25 cm2 thick of aluminum shielding (see below).

I could not find much info on the current neutron rate/dose in space (neutrons decay in 10min) but in the Nelson 2016 paper they posit about 20-30mGy dose over three years.

The total dose expected over a 900 day mission to Mars (~500 days on the planet) has been estimated to be 40 cGy. The deep space dose rate is 1 mGy/day (all sources), which would be cut in half on the surface of Mars due to planetary shielding.

Paper is important as it is one of the first to do long-term exposures.

Thank you and this was in fact the principal driver for these studies.

The KDE is a smoothing function (estimate probability distribution of a random variable) and based on the results don't tell us how they fit the data - they simply grab the top 95% - but sort of poor rationale at least for me - They make some strong assertions based on the KDE - so they need to model this a bit better - details like the bandwidth that they used... there is an optimal (i.e. silvermans) choice. KDE is not good for uniformly distributed data either.

The reviewer is quite correct in that KDE is not good for uniformly distributed data. We did in fact state in the paper that “Given that the distribution of behavior data is gamma distributed, we also applied a non-parametric approach, kernel density estimation (KDE), to calculate the proportion of mice that exhibited impaired performance within each cohort for a given behavior task.” Since Silverman's Rule of Thumb really only works well when the true density is approximately normal, this would be far from optimal in this case.

In the Jewell paper we did cite the algorithm that we use to calculate the KDE, and thought that referencing that paper would be sufficient. We have now added an additional reference so that readers can see exactly what algorithm we used and now state that “Data was analyzed using the Excel add-in and Minitab KDE macro (AMC Kernel density ver 1.0e) available in AMC Software (Royal Society of Chemistry: www.rsc.org/amc/). This algorithm has been design to estimate population density functions without making any assumptions. It does not use a fixed bin width, but instead calculates an optimal bin width based upon the inter-quartile range. Full details can be found in the amc technical brief Analytical Methods Committee No. 4. Jan 2001 Royal Society of Chemistry 2001. https://www.rsc.org/images/data-distributions-kernel-density-technical-brief-4_tcm18-214836.pdf . This algorithm is widely used in industry because of its robustness and is ideally suited to analyzing non-uniformly distributed data populations. Please refer to pg. 14.

We do determine define the performance metric level within the sham cohort that encompasses the top 95% of performers, and thus establish the 5th percentile. We then determine how many of the irradiated mice have performance metrics that fall within the 5th percentile of the sham population performance. If the data set was normally distributed a Z score of 2 would encompass 95% of the data. Most people would agree that a Z-score >2 would be significantly different from the population mean. As stated we used an analogous approach to the KDE data and used the 5th percentile as our definition point of “severely impaired”.

- Ephsyio data looks good, but in some instances the resultant significance appears comes to come from a few animals in each that seem to drive the significance.

We agree that some measures show a notable degree of variability. We utilized the same set of exclusion criteria across all recordings/treatment groups and have no clear justification for removing any of the presented data points. We have now added estimation statistics to our analysis of all the whole cell recording data, providing a nonparametric bootstrapping analysis of the effect sizes we observe.

Overall interesting and important for space travel, but again neutrons are not overly important relative to heavy ions; I think if you decide to keep this manuscript, I would force them to be very specific on which types of neutrons they are modeling and that it is distinct from other GCR particles.

We appreciate your points, but I must re-emphasize that we are not trying to model neutron exposures in space. We can do space radiation simulations of the GCR much more accurately at Brookhaven National Lab, which is the source of 99.99% of the NASA radiation publications to date. Unfortunately, practical limitations prevent the use and operation of this facility for 6 continuous months. Our use of the neutron facility was designed specifically to simulate the prolonged low dose rate environment in space, with the added benefit that the integral neutron energy spectra from 252Cf fission neutrons is most realistic to the energy spectra of neutrons within the spacecraft calculated for a shielding thickness of 25 g/cm2 of aluminum. During a deep space mission to Mars, the total absorbed dose contribution from neutrons has been estimated to be ~10%, and would be derived from all GCR interactions liberating neutrons form the planet surface, all spacecraft components and the body of the astronaut. We have deliberately left the majority of these consideration out of the present manuscript, due to the target audience of this journal. Instead, we have decided to focus on the neurobiological impact of low dose rate radiation effects. If the reviewer and/or Editor think it necessary, we can easily incorporate additional radiation specific detail in the discussion.
